# Protoplast Transformation of Recalcitrant Alkaliphilic *Bacillus* sp. with Methylated Plasmid DNA and a Developed Hard Agar Regeneration Medium

**DOI:** 10.1371/journal.pone.0028148

**Published:** 2011-11-23

**Authors:** Chenghua Gao, Yanfen Xue, Yanhe Ma

**Affiliations:** 1 State Key Laboratory of Microbial Resources, Institute of Microbiology, Chinese Academy of Sciences, Beijing, China; 2 Graduate School of the Chinese Academy of Sciences, Beijing, China; Universidad Nacional Autonoma de Mexico, Instituto de Biotecnologia, Mexico

## Abstract

Among the diverse alkaliphilic *Bacillus* strains, only a little have been reported to be genetically transformed. In this study, an efficient protoplast transformation procedure was developed for recalcitrant alkaliphilic *Bacillus* sp. N16-5. The procedure involved polyethylene glycol-induced DNA uptake by the protoplasts and subsequent protoplast regeneration with a developed hard agar regeneration medium. An in vivo methylation strategy was introduced to methylate the exogenous plasmid DNA for improving the transformation efficiency. The transformation efficiency reached to 1.1×10^5^ transformants per µg plasmid DNA with methylated plasmid pHCMC04 and the developed hard agar regeneration medium. This procedure might also be applicable to the genetic transformation of other *Bacillus* strains.

## Introduction

Alkaliphiles are defined as microorganisms that grow optimally at pH of over 9 [1]. Alkaliphilic *Bacillus* strains are of industrial importance because they produce various alkaline extracellular enzymes exhibiting great values in industrial application [2], [3], [4]. Also alkaliphilic *Bacillus* strains are promising metabolite producers of carotenoids, cholic acid derivatives, and organic acids [5], [6], [7].

For in-depth study of the metabolic mechanisms and physiological alkaline adaptation mechanisms of the alkaliphilic *Bacillus* strains at the molecular level, the development of genetic transformation systems is indispensable. To date, most investigations on physiological alkaline adaptation mechanisms of alkaliphiles are limited in the two facultatively alkaliphilic strains, *Bacillus pseudofirmus* OF4 and *Bacillus halodurans* C-125 [8], [9], which can be genetically transformed [10], [11], [12]. Nevertheless, since the diversity of alkaliphilic *Bacillus* strains are very rich [13] and different strains may adopt distinct strategies to adapt to the external alkaline environment [8], [14], the establishment of genetic transformation systems in other alkaliphilic *Bacillus* strains may be helpful to broaden the study on the physiological alkaline adaptation mechanisms of alkaliphiles. Besides, it can be useful to metabolically engineer these strains for production of industrial valuable metabolites. *Bacillus* sp. N16-5 is a recalcitrant facultatively alkaliphilic strain, producing multiple extracellular hydrolases and organic acids. It is phylogenetically related to *Bacillus agaradhearens* [15] and distant from *Bacillus pseudofirmus* and *Bacillus halodurans* [13]. Although several extracellular hydrolases produced by this strain have been characterized [15], [16], [17], the further research is hindered by the lack of a genetic transformation system. So the aim of this study is to develop a genetic transformation method for *Bacillus* sp. N16-5.

It is well known that the restriction and modification (RM) systems act as “immune” systems in prokaryotes for defending the hosts against the invasion of exogenous DNA [18]. Most RM systems consist of a restriction endonuclease (REase) and a methyltransferase (MTase). The REase cleaves the “foreign” DNA to avoid its intrusion whereas the MTase methylates “self” DNA to resist the digestion of the REase [19]. Three major types of RM systems (type I, II, III) have been identified and characterized based on protein structure, cofactor requirements and recognition sites [20]. In spite of their protective roles, The RM systems degrade the incorporated exogenous DNA and therefore become a genetic barrier for the DNA introduction into the hosts [21], [22], [23], [24]. To relieve the block of RM systems during the DNA introduction, many in vivo methylation strategies have been developed to methylate the exogenous DNA prior to the DNA transfer [25], [26], [27]. The strategy usually contained a methylation plasmid carrying the MTase gene of the target bacterial strain and a shuttle plasmid used for genetic transformation. After co-transformation of the two plasmids into *Escherichia coli* (*E. coli)*, the shuttle plasmid would be methylated by the MTase, which was encoded by the MTase gene from the methylation plasmid.

In this study, an efficient protoplast transformation procedure was introduced for *Bacillus* sp. N16-5 with a developed hard agar regeneration medium. Additionally, an in vivo methylation strategy was exploited to methylate plasmid DNA for improving the transformation efficiency. Prior to the successful protoplast transformation of *Bacillus* sp. N16-5, the method of electroporation transformation [11] was also adopted to transform this strain, but without success.

## Results

### Protoplast formation

The protoplast formation of *Bacillus* sp. N16-5 was a very easy process. Over 99% of the bacillary cells formed a spherical state at the concentration of 0.1 mg/ml of lysozyme after a 30 min treatment at 37°C under the observation of the optical microscopy. But the cells were treated with lysozyme of 0.2 mg/ml at 37°C for 60 min for complete protoplastization, which was necessary for efficient protoplast transformation [28].

### Protoplast regeneration and transformation with a hard gelatin regeneration medium

**Figure 1:**
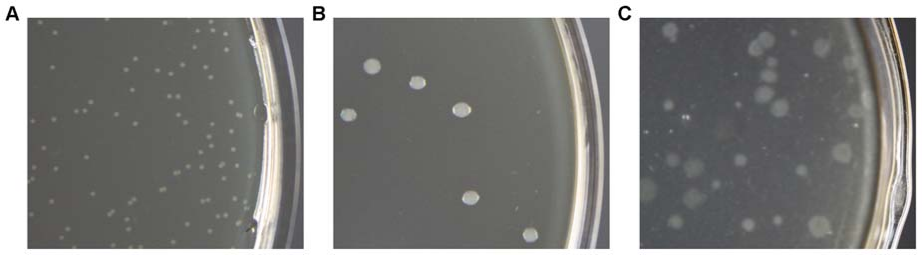
Protoplast regeneration of *Bacillus* sp. N16-5 on different media. **A.** L colonies formed on SA1.2 medium. **B.** regenerated bacillary colonies formed on SG25 medium. **C.** regenerated bacillary colonies formed on SA5 medium.

Succinate medium solidified with 1.2% agar (SA1.2 medium), a soft agar regeneration medium modified from DM3 medium [29], was firstly used to test the protoplast regeneration of *Bacillus* sp. N16-5. After incubation of the protoplasts plated on the medium at 37°C for 5–7 days, only small, round and transparent colonies with a diameter of about 0.5 mm grew up (Figure 1A). However, these colonies could not be transferred to several hypertonic or nonhypertonic media, or even the original SA1.2 medium. They turned out to be L colonies (colonies formed by the bacterial strains that lack cell wall), but not the cell wall regenerated bacillary colonies [30]. 10.1% of the protoplasts plated on the medium formed L colonies. For reversion of the protoplast to the bacillary form, several modifications of SA1.2 medium had been examined, including addition of autoclaved *Bacillus* sp. N16-5 cells [31], substitution of bovine serum albumin (BSA) by maize starch or skim milk [32], replacement of sodium succinate by sucrose [33] or mannitol [34], addition of 2.5% gelatin [34], removal of BSA, and lowering the incubation temperature from 37°C to 25°C. However, neither L colonies nor bacillary colonies arose when sucrose or mannitol was used as the osmotic stabilizer and BSA was substituted by skim milk. And the other trials just still brought about L colonies. The above results indicated that the protoplast of *Bacillus* sp. N16-5 could not regenerate the cell wall on the modified DM3 medium, which could support the successful protoplast regeneration and transformation of alkaliphilic *Bacillus pseudofirmus* OF4 and *Bacillus halodurans* C-125 [10], [12]. According to the previous report, protoplast reversion of *Bacillus subtilis* was stimulated by the close physical contact between the naked protoplasts and the hard substratum provided by the regeneration medium with 25% gelatin, hard agar or membrane filters [31], [35]. So succinate medium solidified with 25% gelatin (SG25 medium), a hard gelatin regeneration medium, was used to stimulate the protoplast regeneration. The protoplasts of *Bacillus* sp. N16-5 successfully formed bacillary colonies after incubation on SG25 medium at 25°C for 8–10 days (Figure 1B). Besides, the L colonies formed on SA1.2 medium could reverse to the bacillary colonies when transferred to SG25 medium. The protoplast regeneration frequency with SG25 medium was 8.4×10^−5^. In this study, protoplast regeneration frequency was calculated by the ratio of the cell wall regenerated bacillary colonies formed on the regeneration medium to the initial protoplasts plated on it.

Protoplast transformation was firstly accomplished by plasmid pHCMC04 and pBK15 with the transformation efficiency of about 10^3^ transformants per µg plasmid DNA. Also plasmid pMK4 and pHP13 could be transformed into *Bacillus* sp. N16-5. All of the four plasmids could be maintained well in *Bacillus* sp. N16-5 at both neutral complex medium (pH 7.7) [11] and Horikoshi-I medium with 2% NaCl (pH 10.0) [1] with appropriate antibiotic.

### Protoplast regeneration with a hard agar regeneration medium to reduce the transformation time

**Figure 2:**
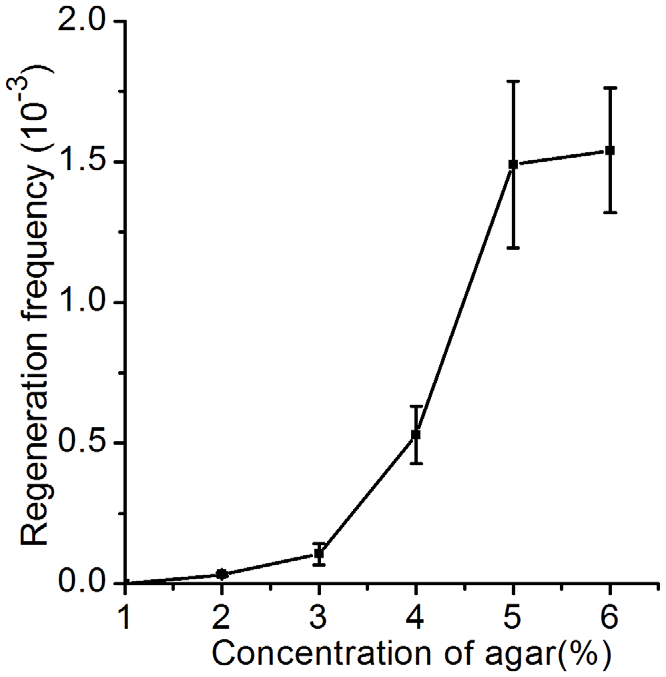
The effect of the agar concentration in SA1.2 medium on the protoplast regeneration frequency. All of the experiments in this study were repeated three times.

Protoplast transformation of *Bacillus* sp. N16-5 was achieved with SG25 medium. However, the protoplast regeneration took about 8 days and therefore became a time-consuming step for the protoplast transformation as a result of the low incubation temperature of the medium (25°C). To shorten the time of protoplast regeneration, we tried to find out a regeneration medium that could be incubated at higher temperature. Previous report found that the regeneration frequency was strongly affected by the agar concentration in the regeneration medium [35]. Protoplast regeneration of *Bacillus subtilis* with the regeneration medium containing 0.9% agar produced a combination of L colonies and bacillary colonies, whereas the medium with 2.5% agar could provide a hard substratum to induce the complete protoplast reversion [35]. So we enhanced the concentration of agar in SA1.2 medium from 1.2% to 2.5%. During the change of the agar concentration in SA1.2 medium, the pH of the medium was adjusted to 8.0 and BSA was omitted in the medium. After incubating the protoplasts of *Bacillus* sp. N16-5 on the adjusted medium at 37°C for 2–3 days, both bacillary colonies and L colonies appeared. However, over 99% of the total colonies were L colonies. The predominance of L colonies suggested that the regeneration medium with 2.5% agar probably could not provide a physical substratum strong enough for the complete protoplast regeneration of *Bacillus* sp. N16-5. So we tried to further increase the concentration of agar in SA1.2 medium to promote the physical strength of the medium and test its effect on the regeneration frequency. As shown in Figure 2, the protoplast regeneration frequency arose with the increment of the agar concentration in SA1.2 medium. Meanwhile, the ratio of L colonies to the bacillary colonies declined with the increment of the agar concentration. Over 95% of the colonies grown on the medium with 5% or 6% agar were bacillary colonies. So succinate medium solidified with 5% agar (SA5 medium), a hard agar regeneration medium, was used for subsequent protoplast regeneration and transformation. The regenerated bacillary colonies formed on SA5 medium were shown in Figure 1C. The regeneration frequency with SA5 medium was 1.5×10^−3^, which was over 10 times higher than that with SG25 medium. What's more, the protoplast regeneration with SA5 medium only needed 2–3 days, compared with about 8 days with SG25 medium.

**Figure 3:**
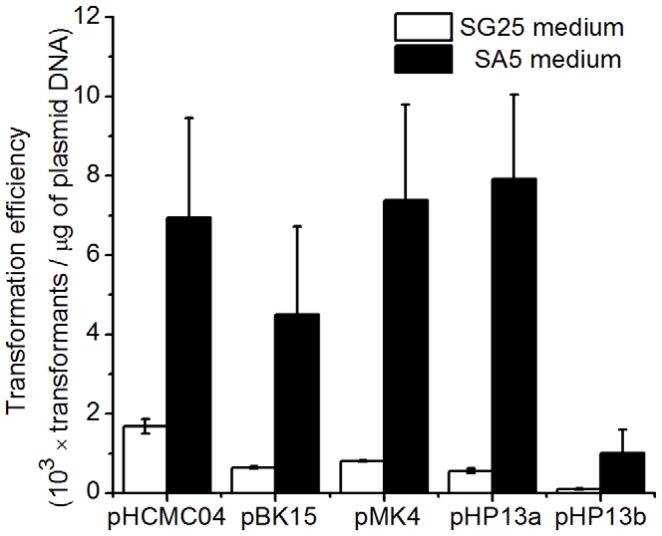
Protoplast transformation efficiency of different plasmids with SG25 medium and SA5 medium. pHP13a, transformation of plasmid pHP13 using chloramphenicol as the selective antibiotic; pHP13b, transformation of plasmid pHP13 using erythromycin as the selective antibiotic.

Protoplast transformation was carried out with SA5 medium. The transformation efficiency of different plasmids with SA5 medium was about 4–10 times higher than that with SG25 medium (Figure 3), which probably resulted from the much higher regeneration frequency with SA5 medium. With the protoplast transformation procedure developed for *Bacillus* sp. N16-5, another recalcitrant facultatively alkaliphilic strain, *Bacillus agaradhearens* DSM 871, was successfully transformed by the above four plasmids with the transformation efficiency of about 10^3^ transformants per µg plasmid DNA using SG25 medium and about 10^5^ transformants per µg plasmid DNA using SA5 medium.

### Construction of an in vivo methylation strategy

Although we had successfully shortened the transformation time and improved the transformation efficiency by developing SA5 medium as the regeneration medium, the transformation efficiency of about 10^4^ transformants per µg plasmid DNA was still very low. So we tried to further improve the transformation efficiency. During the genetic transformation of the bacterial strains that possessed the RM system, the transformation efficiency was greatly improved by the employment of methylated plasmid DNA that could resist the digestion of the restriction enzymes in the bacterial strains [26], [27]. So we tried to identify whether there was a RM system existing in *Bacillus* sp. N16-5 that could prevent the incorporation of the exogenous unmodified plasmid DNA and therefore reduce the transformation efficiency.

**Figure 4:**
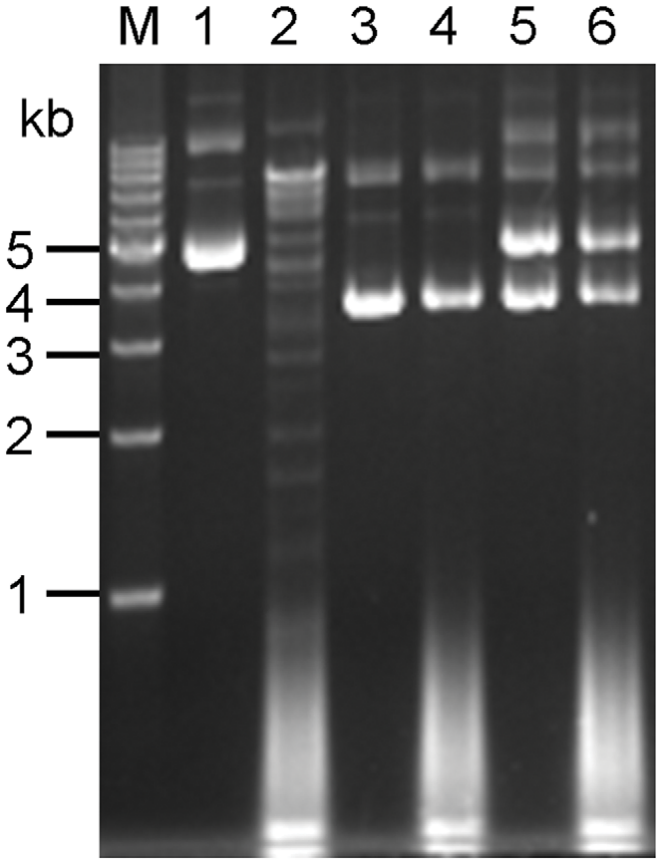
Detection of the REase activity in *Bacillus* sp. N16-5 and the effect of the in vivo methylation strategy. *Lane M*, molecular weight marker; *lane 1*, unmethylated plasmid pHCMC04; *lane 2*, unmethylated plasmid pHCMC04 digested by the whole cell extract; *lane 3*, plasmid pAM1; *lane 4*, plasmid pAM1 digested by the whole cell extract; *lane 5*, methylated plasmid pHCMC04; *lane 6*, methylated plasmid pHCMC04 digested by the whole cell extract. The smeared DNA bands (<1 kb) in *lane 2*, *4* and *6* indicated the genomic DNA fragments in the whole cell extract generated by the sonication.

To detect the existence of the RM system in *Bacillus* sp. N16-5, unmodified plasmid pHCMC04 was treated with the whole cell extract prepared from *Bacillus* sp. N16-5. As shown in Figure 4, unmodified plasmid pHCMC04 was digested into a discrete DNA fragment pattern by the whole cell extract, which indicated the presence of a site-specific REase in this strain [36]. To determine the cleavage site of the REase, plasmid pUC18 and pMK4 were separately digested by the whole cell extract with the resulted DNA fragments sequenced. The sequence alignment of the DNA fragments with plasmid pUC18 or pMK4 revealed only one kind of cleavage site with a palindromic sequence of 5′-CGCG-3′, which suggested there was a type II REase in *Bacillus* sp. N16-5 [20]. The presence of the type II REase might obstruct the protoplast transformation of plasmid DNA. So an in vivo methylation strategy was developed to methylate exogenous plasmid DNA and protect them from the digestion of the type II REase. Plasmid pAM1, harboring the type II MTase gene of *Bacillus* sp. N16-5, was constructed. For methylation of plasmid pHCMC04, plasmid pAM1 and pHCMC04 were co-transformed into *Escherichia coli* (*E. coli*) Top10. The mixture of plasmid pAM1 and pHCMC04 was extracted and digested by the whole cell extract to determine the effect of the in vivo methylation strategy. As shown in Figure 4, the mixture of plasmid pAM1 and pHCMC04 resisted to the digestion of the type II REase in the whole cell extract, which indicated that plasmid pHCMC04 and pAM1 were both successfully methylated by the in vivo methylation strategy.

### Improving the protoplast transformation efficiency by methylated plasmids

**Figure 5:**
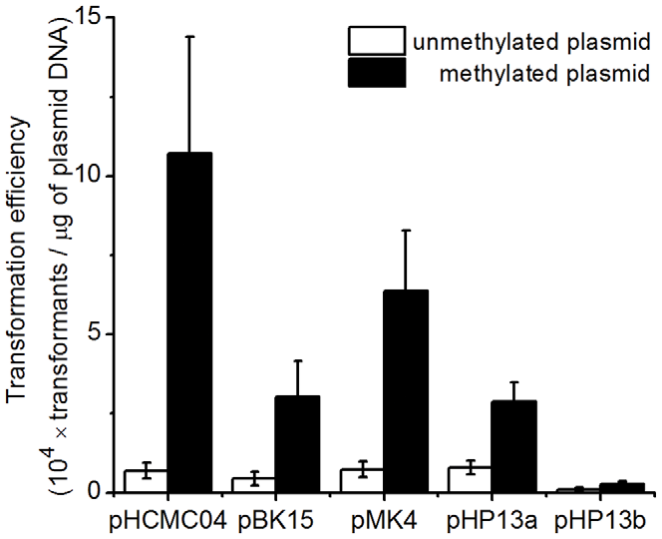
Protoplast transformation efficiency of unmethylated and methylated plasmids with SA5 medium.

Protoplast transformation was carried out with methylated plasmids and SA5 medium. The methylated plasmid DNA was transformed with higher efficiency than the unmethylated, and the transformation efficiency of the methylated plasmid pHCMC04, pBK15 and pMK4 was about 10 times higher than that of the unmethylated (Figure 5). This result indicated that the in vivo methylation strategy successfully promoted the transformation efficiency by methylating plasmid DNA and protecting them against the REase of *Bacillus* sp. N16-5. The highest transformation efficiency of 1.1×10^5^ transformants per µg plasmid DNA was achieved with methylated plasmid pHCMC04 and SA5 medium.

## Discussion

Protoplast regeneration was the major difficult step during the protoplast transformation of *Bacillus* strains, and L colonies often emerged on the soft agar regeneration medium during the protoplast regeneration [30], [35], [37], [38]. Modifications of the soft agar regeneration medium for the successful protoplast regeneration, including addition of cell wall fractions or autoclaved intact cells, alternation of the osmotic stabilizer, change of minerals, and addition of polyvinyl pyrrolidone, only gave inconsistent results [30], [38]. The physical property of the regeneration medium greatly influenced the protoplast regeneration frequency [35], [39], and the hard regeneration medium with 25% gelatin or 25% pluronic polyol F127 might provide a more general condition for stimulating the protoplast regeneration of diverse *Bacillus* stains [37], [38]. However, due to the low melt point of gelatin (28°C), the protoplast regeneration with the hard gelatin regeneration medium became a time-consuming process and could not apply to the protoplast regeneration of thermophilic *Bacillus* strains, which needed high temperature [38], [40]. And as pluronic polyol F127 only gelled well at high temperature and became unstable at low temperature (below 30°C) [38], [41], the hard regeneration medium with 25% pluronic polyol F127 might be unsuitable for the protoplast regeneration of *Bacillus* strains that particularly needed low temperature. Here we developed a hard agar regeneration medium (SA5 medium), which could provide a rather strong substratum for efficient protoplast regeneration of *Bacillus* sp. N16-5. Furthermore, it was stable at a much broader range of temperature (below 60°C). So SA5 medium might be helpful for the protoplast regeneration and transformation of a greater range of *Bacillus* stains.

In this study, we successfully identified a type II RM system in *Bacillus* sp. N16-5 and developed an in vivo methylation strategy to methylate the *E. coli-Bacillus* sp. shuttle plasmids. Unlike some previous in vivo methylation strategy in which the MTase was expressed by an exogenous promoter (*araBAD* promoter) [26], [27], the MTase expressed by the native promoter of its gene was sufficient to methylate the shuttle plasmids in this study. So it suggested that the exogenous promoter was not prerequisite to express the MTase in *E. coli* for plasmid methylation and the native promoter of the MTase gene might be qualified in an in vivo methylation strategy [25].

This paper described a quick, efficient and highly reproducible procedure for protoplast transformation of recalcitrant alkaliphilic *Bacillus* sp. N16-5 with the combination of a newly developed hard agar regeneration medium (SA5 medium) and an in vivo methylation strategy. Also the procedure could be used to transform recalcitrant alkaliphilic *Bacillus agaradhearens* DSM 871 with unmodified plasmid DNA. The method could be helpful for gene inactivation in *Bacillus* sp. N16-5 by chromosome integration with the thermosensitive plasmid [42] or Campbell-like recombination [43] in the future. Moreover, this method might be applicable to the genetic transformation of other alkaliphilic or even neutralophilic *Bacillus* strains.

## Materials and Methods

### Bacterial strains, plasmids, media, and cultural conditions

Bacterial strains and plasmids used in this study were showed in Table 1.

**Table 1:** Bacterial strains and plasmids.

Strains or plasmids	Descriptions	Reference or source
Strains		
*E. coli* Top10	*mcrA Δ(mrr-hsdRMS-mcrBC) recA1*	Invitrogen
*Bacillus* sp. N16-5	A facultative alkaliphilic strain, producing multiple extracellular hydrolases and organic acids	Lab storage
*Bacillus agaradhaerens* DSM 8721	A facultative alkaliphilic strain	DSMZ
Plasmids		
pUC18	2.7 kb, Amp^R^	Invitrogen
pAN2	6.8 kb, Φ*3t*I, p15A origin, Tet^R^	[46]
pAM1	6.3 kb, *bsp165IM*, p15A origin, Tet^R^, methylating plasmid DNA prior to transformation to protect it against the restriction system of *Bacillus* sp. N16-5	This study
pHCMC04	8.1 kb; Amp^R^, ColE1 origin in *E. coli*; Cm^R^, pBS72 origin in *Bacillus* sp.	[47]
pBK15	6.6 kb; Amp^R^, ColE1 origin in *E. coli*; Cm^R^, pE194 origin in *Bacillus* sp.	[10]
pMK4	5.6 kb; Amp^R^, Cm^R^, ColE1 origin in *E. coli*; Cm^R^, pC194 origin in *Bacillus* sp.	[48]
pHP13	4.9 kb; Em^R^, Cm^R^, ColE1 origin in *E. coli*; Em^R^, Cm^R^, pTA1060 origin in *Bacillus* sp.	[49]

Abbreviations: Amp^R^, ampicillin resistance; Cm^R^, chloramphenicol resistance; Tet^R^, tetracycline resistance; Em^R^, erythromycin resistance; Φ*3t*I, Φ3TI methyltransferase gene of *Bacillus subtilis* phage Φ3TI; *bsp165IM*, the type II DNA methyltransferase gene of *Bacillus* sp. N16-5; DSMZ, German Collection of Microorganisms and Cell Cultures, Braunschweig, Germany.

SA1.2 medium (pH 7.5) consisted of 1.2% agar, 0.5 M sodium succinate, 30 mM Tris base, 0.5% casamino acid, 0.5% yeast extract, 30 mM MgCl_2_, 12.5 mM CaCl_2_, 1% NaCl, 0.5% glucose and 0.04% filter-sterilized bovine serum albumin. SG25 medium (pH 8.0), which was modified from GR medium [44] and DM3 medium, contained 25% gelatin, 0.3 M sodium succinate, 40 mM Tris base, 0.5% casamino acid, 0.5% yeast extract, 30 mM MgCl_2_, 12.5 mM CaCl_2_, 1% NaCl, and 0.5% glucose. SA5 medium (pH 8.0) contained the same components as SA1.2 medium except that the concentration of agar and Tris base was adjusted to 5% and 10 mM and BSA was omitted in SA5 medium. SMM buffer (pH 7.0) contained 0.5 M sucrose, 0.02 M sodium maleate and 0.02 M MgCl_2_. SMMP buffer (pH 7.0) was prepared by mixing equal volumes of separately sterilized 2×strength SMM and 4×strength Penassay broth. 30% polyethylene glycol (PEG) 8000 was dissolved in SMM buffer.

*E. coli* Top10 was grown aerobically at 37°C in Luria-Bertani medium supplemented, when necessary, with ampicillin (100 µg/ml), tetracycline (40 µg/ml), chloramphenicol (30 µg/ml), or erythromycin (150 µg/ml). *Bacillus* sp. N16-5 was grown aerobically at 37°C in Horikoshi-I medium with 2% NaCl (pH 10.0) or complex neutral medium (pH 7.7) supplemented, when necessary, with chloramphenicol (2.5 µg/ml), or erythromycin (1 µg/ml). All strains were maintained frozen in 25% glycerol at −80°C.

### DNA manipulation

The genomic DNA preparation, plasmid DNA extraction and DNA purification were carried out with Genomic DNA Extraction Kit (Sangon, Shanghai, China), E.Z.N.A Plasmid Extraction Kit (Omega Biotek Inc., Guangzhou, China) and E.Z.N.A Gel Extraction Kit (Omega Biotek Inc., Guangzhou, China) following the manufacturer's instructions. For plasmid extraction from *Bacillus* sp. N16-5, the bacterial pellet was pretreated with lysozyme of 2 mg/ml at 37°C for 10 min prior to the standard steps of E.Z.N.A Plasmid Extraction Kit. DNA restriction and cloning were carried out according to the standard procedures [45]. *Pyrobest* DNA polymerase, restriction enzymes, T4 DNA ligase, T4 DNA polymerase and pMD18-T vector were purchased from Takara (Dalian, China). DreamTaq DNA polymerase was purchased form Fermentas (Beijing, China).

### Protoplast preparation and regeneration

A 1% inoculum of the overnight culture of *Bacillus* sp. N16-5 in Horikoshi-I medium with 2% NaCl was inoculated into 100 ml neutral complex medium. After incubation at 37°C, 220 rpm for about 5 h, the culture reached to the early log growth phase with an OD600 of 0.4. Then the culture was collected by centrifugation at 4°C and 5 000×g for 5 min. The collection was washed once with 10 ml ice-cooled SMMP and suspended in 10 ml SMMP. Lysozyme was added to the suspension with a final concentration of 0.2 mg/ml and mixed by gently shaking. The suspension was incubated at 37°C for 60 min. Then 10 ml SMMP was mixed with the suspension before the resulted protoplasts were harvested by centrifugation. The protoplast collection was washed once with 10 ml SMMP and suspended in 2.5 ml SMMP. The suspension was allocated with 100 µl per aliquot and stored at −80°C. To detect the protoplast regeneration, the prepared protoplasts were plated onto the regeneration medium and incubated at 25°C for SG25 medium or at 37°C for the other media.

### Protoplast transformation

100 µl of the prepared protoplast suspension was added with 4 µl of plasmid DNA solution (0.5–1 µg DNA) followed by mixing with 300 µl of 30% PEG 8000 solution. After 3 min incubation at room temperature, the protoplast suspension was mixed gently with 5 ml SMMP. Then the protoplast was recovered by centrifugation at 4°C and 5 000×g for 10 min and suspended in 1 ml SMMP. For expression of the chloramphenicol or erythromycin resistance gene, the suspension was incubated at 28°C and 120 rpm for 2 h. To select the transformants, the incubated suspension was plated onto the regeneration medium (SG25 medium or SA5 medium) supplemented with chloramphenicol (2.5 µg/ml), or erythromycin (0.5 µg/ml).

### Preparation of the whole cell extract for detection of the REase in *Bacillus* sp. N16-5

*Bacillus* sp. N16-5 was grown in 50 ml Horikoshi-I medium to reach to an OD600 of 2.0. Then the culture was collected by centrifugation, washed once with 50 mM K_2_HPO_4_-KH_2_PO_4_ buffer (pH 7.5) and suspended in 5 ml of the buffer. The suspension was lysed by sonication at 150 W for 25 min and centrifuged at 4°C and 15 000×g for 20 min. The supernatant was stored as the whole cell extract. The whole cell extract was used for the detection of the REase in *Bacillus* sp. N16-5. The reaction system of the detection contained 1 µg plasmid DNA, 8 µl of the whole cell extract and 2 µl 10×Takara buffer H (Takara, Dalian, China) in a total volume of 20 µl. And the reaction was performed at 37°C for 8 h.

### Determination of the cleavage site of the REase in *Bacillus* sp. N16-5

Plasmid pUC18 and pMK4 were separately digested by the whole cell extract of *Bacillus* sp. N16-5. The resulted DNA fragments were harvested by gel extraction. The recovered DNA fragments were blunt-ended by T4 DNA polymerase and added with an adenine nucleotide tailing by DreamTaq DNA polymerase. Then the modified DNA fragments were ligated with pMD18-T vector for sequencing. The sequences of DNA fragments were aligned with the sequence of plasmid pUC18 or pMK4 to locate the REase cleavage site.

### Construction of an in vivo methylation strategy

*E. coli* Top10 was used for construction of plasmid pAM1. According to the genome data of *Bacillus* sp. N16-5 (unpublished data), the gene that might encode a type II DNA MTase (Genbank number: JN381161) was selected and named as *bsp165IM*. The primer MetEcoRIF (5′-CCGGAATTCCGAATTGTTGCATTTCTTGAT-3′) and MetNdeIR (5′-CAGCCATATGCCAGCCTCTTGCAATTATTAT-3′) were used to amplify the *bsp165IM* gene together with its own promoter sequence (the underlined letters indicated the restriction enzyme recognition sites). The PCR product was digested by EcoRI and NdeI, and plasmid pAN2 was also digested by EcoRI and NdeI to remove the initial Φ3TI methyltransferase gene. These two digested products were ligated together to generate plasmid pAM1. Plasmid pAM1 had a p15A origin of replication, which was compatible with the ColE1 origin of plasmid pHCMC04, pBK15, pMK4 and pHP13 in *E. coli*. For methylating the plasmid pHCMC04, plasmid pHCMC04 and pAM1 were co-transformed into *E. coli* Top10 and maintained in it with ampicillin and tetracycline. The other plasmids were methylated as the same method above with appropriate antibiotics.

## References

[1] Horikoshi K (1999). Alkaliphiles: some applications of their products for biotechnology.. Microbiol MoL Biol Rev.

[2] Horikoshi K (1971). Production of alkaline enzymes by alkalophilic microorganisms. I. Alkaline protease produced by *Bacillus* No. 221.. Agric Biol Chem.

[3] Ito S, Kobayashi T, Ara K, Ozaki K, Kawai S (1998). Alkaline detergent enzymes from alkaliphiles: enzymatic properties, genetics, and structures.. Extremophiles.

[4] Fujinami S, Fujisawa M (2010). Industrial applications of alkaliphiles and their enzymes - past, present and future.. Environmental Technology.

[5] Aono R, Horikoshi K (1991). Carotenes produced by alkaliphilic yellow pigmented strains of *Bacillus*.. Agric Biol Chem.

[6] Kimura H, Okamura A, Kawaide H (1994). Oxidation of 3-, 7-, and 12-hydroxyl groups of cholic acid by an alkalophilic *Bacillus* sp.. Biosci Biotechnol Biochem.

[7] Paavilainen S, Helistö P, Korpela T (1994). Conversion of carbohydrates to organic acids by alkaliphilic bacilli J Ferment Bioeng.

[8] Padan E, Bibi E, Ito M, Krulwich TA (2005). Alkaline pH homeostasis in bacteria: new insights.. Biochim Biophys Acta.

[9] Krulwich TA, Sachs G, Padan E (2011). Molecular aspects of bacterial pH sensing and homeostasis.. Nat Rev Microbiol.

[10] Ito M, Guffanti AA, Zemsky J, Ivey DM, Krulwich TA (1997). Role of the nhaC-encoded Na+/H+ antiporter of alkaliphilic *Bacillus firmus* OF4.. J Bacteriol.

[11] Ito M, Nagane M (2001). Improvement of the electro-transformation efficiency of facultatively alkaliphilic *Bacillus pseudofirmus* OF4 by high osmolarity and glycine treatment.. Biosci Biotechnol Biochem.

[12] Kudo T, Hino M, Kitada M, Horikoshi K (1990). DNA sequences required for the alkalophily of *Bacillus* sp. strain C-125 are located close together on its chromosomal DNA.. J Bacteriol.

[13] NieIsen P, Fritze D, Priest FG (1995). Phenetic diversity of alkaliphilic *Bacillus* strains: proposal for nine new species.. Microbiology.

[14] Gilmour R, Messner P, Guffanti AA, Kent R, Scheberl A (2000). Two-dimensional gel electrophoresis analyses of pH-dependent protein expression in facultatively alkaliphilic *Bacillus pseudofirmus* OF4 lead to characterization of an S-layer protein with a role in alkaliphily.. J Bacteriol.

[15] Ma Y, Xue Y, Dou Y, Xu Z, Tao W (2004). Characterization and gene cloning of a novel beta-mannanase from alkaliphilic *Bacillus* sp. N16-5.. Extremophiles.

[16] Li G, Rao L, Xue Y, Zhou C, Zhang Y (2010). Cloning, expression, and characterization of a highly active alkaline pectate lyase from alkaliphilic *Bacillus* sp. N16-5.. J Microbiol Biotechnol.

[17] Zhang G, Mao L, Zhao Y, Xue Y, Ma Y (2010). Characterization of a thermostable xylanase from an alkaliphilic *Bacillus* sp.. Biotechnol Lett.

[18] Nikolajewa S, Beyer A, Friedel M, Hollunder J, Wilhelm T (2005). Common patterns in type II restriction enzyme binding sites.. Nucleic Acids Res.

[19] Wilson GG, Murray NE (1991). Restriction and modification systems.. Annu Rev Genet.

[20] Yuan R (1981). Structure and mechanism of multifunctional restriction endonucleases.. Ann Rev Biochem.

[21] Lorenz MG, Wackernagel W (1994). Bacterial gene transfer by natural genetic transformation in the environment.. Microbiol Rev.

[22] Elhai J, Vepritskiy A, Muro-Pastor AM, Flores E, Wolk CP (1997). Reduction of conjugal transfer efficiency by three restriction activities of *Anabaena* sp. strain PCC 7120.. J Bacteriol.

[23] Berndt C, Meier P, Wackernagel W (2003). DNA restriction is a barrier to natural transformation in *Pseudomonas stutzeri* JM300.. Microbiology.

[24] Thomas CM, Nielsen KM (2005). Mechanisms of, and barriers to, horizontal gene transfer between Bacteria.. Nat Rev Micro.

[25] Mermelstein LD, Papoutsakis ET (1993). In vivo methylation in *Escherichia coli* by the *Bacillus subtilis* phage phi 3T I methyltransferase to protect plasmids from restriction upon transformation of *Clostridium acetobutylicum* ATCC 824.. Appl Environ Microbiol.

[26] Yasui K, Kano Y, Tanaka K, Watanabe K, Shimizu-Kadota M (2008). Improvement of bacterial transformation efficiency using plasmid artificial modification.. Nucleic Acids Res.

[27] Wallace JG, Breaker RR (2011). Improved genetic transformation methods for the model alkaliphile *Bacillus halodurans* C-125.. Lett Appl Microbiol.

[28] Akamatsu T, Sekiguchi J (1982). Transformation of *Bacillus* Protoplasts by Plasmid pTP4 DNA.. Agric Biol Chem.

[29] Chang S, Cohen SN (1979). High frequency transformation of *Bacillus subtilis* protoplasts by plasmid DNA.. Mol Gen Genet.

[30] Mallonee DH, Speckman RA (1989). Transformation of *Bacillus polymyxa* with plasmid DNA.. Appl Environ Microbiol.

[31] Clive D, Landman OE (1970). Reversion of *Bacillus subtilis* protoplasts to the bacillary form induced by exogenous cell wall, bacteria and by growth in membrane filters.. J Gen Microbiol.

[32] Bhatt YB, Subramanyam VR, Dias FF (1987). The use of starch and skim milk in the regeneration of *Bacillus amyloliquefaciens* and *Bacillus subtilis* protoplasts.. Lett Appl Microbiol.

[33] Puyet A, Sandoval H, López P, Aguilar A, Martín JF (1987). A simple medium for rapid regeneration of *Bacillus subtilis* protoplasts transformed with plasmid DNA.. FEMS Microbiol Lett.

[34] Nimi O, Kubo M, Sugiyama M (1983). Protoplast formation and the regeneration of *Bacillus brevis* ATCC 9999 and its mutants.. Biotechnol Lett.

[35] Landman OE, Halle S (1963). Enzymically and physically induced inheritance changes in *Bacillus subtilis*.. J Mol Biol.

[36] Whitehead PR, Brown NL (1985). A simple and rapid method for screening bacteria for type II restriction endonucleases: enzymes in *Aphanothece halophytica*.. Arch Microbiol.

[37] Bourne N, Dancer BN (1986). Regeneration of protoplasts of *Bacillus subtilis* 168 and closely related strains.. J Gen Microbiol.

[38] Dunn RM, Munster MJ, Sharp RJ, Dancer BN (1987). A novel method for regenerating the protoplasts of thermophilic bacili.. Arch Microbiol.

[39] Mercenier A, Chassy BM (1988). Strategies for the development of bacterial transformation systems.. Biochimie.

[40] Imanaka T, Fujii M, Aramori I, Aiba S (1982). Transformation of *Bacillus stearothermophilus* with plasmid DNA and characterization of shuttle vector plasmids between *Bacillus stearothermophilus* and *Bacillus subtilis*.. J Bacteriol.

[41] Gardener S, Jones JG (1984). A new solidifying agent for culture media which liquefies on cooling.. J Gen Microbiol.

[42] Biswas I, Gruss A, Ehrlich SD, Maguin E (1993). High-efficiency gene inactivation and replacement system for gram-positive bacteria.. J Bacteriol.

[43] Leenhouts KJ, Kok J, Venema G (1989). Campbell-like integration of heterologous plasmid DNA into the chromosome of *Lactococcus lactis* subsp. *lactis*.. Appl Environ Microbiol.

[44] Landman OE, Ryter A, Frehel C (1968). Gelatin-induced reversion of protoplasts of *Bacillus subtilis* to the bacillary form: electron-microscopic and physical study.. J Bacteriol.

[45] Sambrook J, Russell D (2001). Molecular cloning: a laboratory manual: CSHL Press.

[46] Heap JT, Pennington OJ, Cartman ST, Carter GP, Minton NP (2007). The ClosTron: a universal gene knock-out system for the genus *Clostridium*.. J Microbiol Methods.

[47] Nguyen HD, Nguyen QA, Ferreira RC, Ferreira LC, Tran LT (2005). Construction of plasmid-based expression vectors for *Bacillus subtilis* exhibiting full structural stability.. Plasmid.

[48] Sullivan MA, Yasbin RE, Young FE (1984). New shuttle vectors for *Bacillus subtilis* and *Escherichia coli* which allow rapid detection of inserted fragments.. Gene.

[49] Haima P, Bron S, Venema G (1987). The effect of restriction on shotgun cloning and plasmid stability in *Bacillus subtilis*.. Marburg Mol Gen Genet.

